# Supplementary material for: Therapeutic Approaches in Pulmonary Arterial Hypertension with Beneficial Effects on Right Ventricular Function—Preclinical Studies
Source: Int J Mol Sci. 2023 Oct 24;24(21):15539. doi: 10.3390/ijms242115539 (PMC10647677; doi:10.3390/ijms242115539)
Supplement: Supplementary file 1 [file ijms-24-15539-s001.zip › ijms-2665207-supplementary.pdf]

Table S1. Other results.

		RVEF				mPAP				RV/BW				RV weight				RV/TL			
**Study**	Drug(s)	C	M	S	P	C	M	S	P	C	M	S	P	C	M	S	P	C	M	S	P
**1**	Urocortin-2		↑		↑ *																
**2**	Sacubitril/valsartan			↔	↔							↓	↔								
**3**	GS-444217		↑				↓	↓											↓		
**4**	Sodium valproate										↓		↓								
**5**	Ivabradine																				
**6**	Sunitinib																				
**6**	Sorafenib																				
**7**	Juglone						↓										↔				
**8**	Celastrol																				
**9**	Dapaglifozine																				
**10**	Macitentan																				
**10**	Tadalafil																				
**10**	Mac. + Tad.																				
**11**	Neuregulin-1		↑																↓		
**12**	GapmeR H19																				
**13**	MitoQ																				
**14**	Dichloroacetate																				
**15**	Gallein																				
**16**	Sildenafil														↓		↔				
**17**	Clorgyline																				
**18**	Dantrolene										↓										
**19**	RVX208						↓	↔													

mPAP: mean pulmonary arterial pressure; RV: right ventricle; RV/BW: right ventricle weight/bodyweight; RVEF: right-ventricular ejection fraction; RV/TL: right ventricle weight/ tibial length; C: chronic hypoxia; M: monocrotaline (with or without shunt); S: Sugen 5416/hypoxia; P: pulmonary artery banding

↓ - significant decrease in the parameter

↑ - significant increase in the parameter

↔ - no significant effect in the parameter

* - in the animal model, the parameter did not significantly change

Table S2. Other results.

		SV				TIMP-1				Treadmill distance				RVWT				RV FAC			
**Study**	Drug(s)	C	M	S	P	C	M	S	P	C	M	S	P	C	M	S	P	C	M	S	P
**1**	Urocortin-2										↑										
**2**	Sacubitril/valsartan			↔	↔																
**3**	GS-444217						↓	↓													
**4**	Sodium valproate														↓		↓				
**5**	Ivabradine		↑	↑	↑						↑	↔	↑ *						↔	↑	↑
**6**	Sunitinib														↓		↓				
**6**	Sorafenib														↓		↓				
**7**	Juglone																				
**8**	Celastrol											↑					↓			↑	↑
**9**	Dapaglifozine																				
**10**	Macitentan																↔				
**10**	Tadalafil																↔				
**10**	Mac. + Tad.																↔				
**11**	Neuregulin-1																				
**12**	GapmeR H19		↑		↑														↑		↑
**13**	MitoQ													↓			↔				
**14**	Dichloroacetate														↓						
**15**	Gallein										↔		↑								
**16**	Sildenafil		↑		↔		↓		↔												
**17**	Clorgyline			↔	↔																
**18**	Dantrolene														↓						
**19**	RVX208				↑																

SV: stroke volume; RVWT: right-ventricular wall thickness; RV FAC: right-ventricular fractional area change; TIMP-1: mRNA expression of tissue inhibitor of metalloproteinase 1; M: monocrotaline (with or without shunt); S: Sugen 5416/hypoxia;P: pulmonary artery banding

↓ - significant decrease in the parameter

↑ - significant increase in the parameter

↔ - no significant effect in the parameter

* - in the animal model, the parameter did not significantly worsen
